# Association between serum uric acid and muscle function in underweight young women: An exploratory cross-sectional study

**DOI:** 10.1371/journal.pone.0355968

**Published:** 2026-08-14

**Authors:** Shinobu Sugihara, Ichiro Hisatome

**Affiliations:** 1 Matsue Health Service Center, Shimane University, Matsue, Japan; 2 Matsue City Hospital, Matsue, Japan; 3 National Hospital Organization Yonago Medical Center, Yonago, Japan; Seirei Christopher University, JAPAN

## Abstract

**Introduction:**

Uric acid (UA), a major endogenous antioxidant involved in oxidative stress regulation and energy metabolism, has been linked to skeletal muscle health. However, evidence regarding its association with muscle function and muscle mass in underweight young women is limited. This study investigated the associations between serum UA levels, handgrip strength, and skeletal muscle mass in underweight young women.

**Methods:**

This cross-sectional study included 73 underweight female university students (body mass index <18.5 kg/m²) who underwent detailed assessments following annual health checkups at Shimane University, Japan, between 2022 and 2024. Participants were categorized by muscle mass and handgrip strength status based on the Asian Working Group for Sarcopenia (AWGS) 2019 cut-off values. Serum UA levels were compared among groups. Associations between UA and muscle-related parameters were examined using correlation analyses. Multivariable linear regression analyses were performed with handgrip strength as the dependent variable and serum UA as the independent variable, adjusting for age, skeletal muscle index (SMI), estimated glomerular filtration rate (eGFR), daily energy intake, and physical activity.

**Results:**

Serum UA was positively correlated with handgrip strength (r = 0.273, p = 0.013) but not with SMI (r = 0.080, p = 0.503). Participants with low muscle mass and reduced handgrip strength had significantly lower UA levels than those with low muscle mass only (3.4 ± 0.7 vs. 4.1 ± 0.6 mg/dL, p = 0.020). In multivariable linear regression analyses, serum UA was independently associated with handgrip strength after adjustment for age, SMI, eGFR, daily energy intake, and physical activity.

**Conclusions:**

Serum UA was associated with handgrip strength but not with skeletal muscle mass in underweight young women. These findings suggest that serum UA may be more closely related to muscle function than muscle mass in this population. Further studies are needed to clarify the mechanisms underlying this association.

## Introduction

Uric acid (UA), an end product of purine metabolism with antioxidant properties, has been associated with muscle-related outcomes, including muscle mass and muscle strength, particularly among middle-aged and older adults [1–3]. Notably, stronger associations between UA and muscle-related outcomes have been observed in individuals with lower body mass index (BMI) [4]. However, findings have been inconsistent, and a recent Mendelian randomization study did not support a causal relationship between serum UA levels and muscle-related traits [5]. These findings suggest that UA may reflect underlying metabolic or oxidative processes rather than act as a direct causal factor. Therefore, the role of UA in skeletal muscle health remains incompletely understood.

Among the various components of muscle health, muscle strength is increasingly recognized as a more clinically relevant indicator than muscle mass alone. Age-related decline in muscle health has been widely investigated within the framework of sarcopenia, and international consensus groups [6,7], including the European Working Group on Sarcopenia in Older People 2 (EWGSOP2) and the Asian Working Group for Sarcopenia (AWGS), have proposed diagnostic criteria that place greater emphasis on muscle strength than muscle mass [8,9]. Furthermore, the recently updated AWGS 2025 consensus has shifted its focus from sarcopenia to broader muscle health [10].

Although sarcopenia has traditionally been considered an age-related condition, it has also been reported in younger populations [11]. Early muscle impairment in young adults has been associated with lifestyle- and health-related factors such as insufficient energy and protein intake, physical inactivity, chronic illness, and hormonal imbalance [12]. Large-scale epidemiological studies have reported that approximately 10% of individuals in their 20s–30s exhibit sarcopenia-related characteristics, with a higher prevalence observed in Asian populations [13,14]. In Japan, the KEIJI-U study of first-year university students reported a low prevalence of AWGS-defined sarcopenia (approximately 1%); however, underweight individuals were relatively common (16–17%) and exhibited significantly lower skeletal muscle mass and handgrip strength than their normal-weight counterparts [15]. Nevertheless, the sarcopenia-related diagnostic criteria currently applied to young adults were originally developed for older populations, and their applicability to younger individuals remains uncertain [8,9].

Among young adults, underweight women may represent a particularly vulnerable population with respect to muscle health because of chronically low energy availability, reduced fat and muscle reserves, and potential hormonal disturbances associated with menstrual abnormalities [11]. Despite the relatively high prevalence of underweight status among young women in Japan, little is known about the relationship between serum UA and muscle-related parameters in this population. Therefore, this study investigated the associations between serum UA and muscle-related parameters, including handgrip strength and skeletal muscle mass, in underweight female university students.

## Methods

This cross-sectional study was conducted at Shimane University, Japan, between 2022 and 2024. Underweight female university students (BMI < 18.5 kg/m²) were identified through routine health checkups and referred to the university Health Service Center for further assessment. Eligible students were invited to participate in the study. A total of 93 underweight female students were enrolled. Of these, 20 were excluded because blood test data were unavailable, leaving 73 participants for the final analysis.

### Measurements

Participants underwent physical measurements, including blood pressure, heart rate, height, and weight, from which BMI was calculated. Body composition was assessed using a multifrequency bioelectrical impedance analyzer (InBody 240; InBody Japan, Tokyo, Japan). Skeletal muscle mass (SMM), skeletal muscle index (SMI), and body fat percentage were obtained. SMI was calculated as appendicular skeletal muscle mass divided by height squared (kg/m²). Handgrip strength was measured bilaterally using a digital hand dynamometer (Grip-D; Takei Scientific Instruments, Niigata, Japan), and the mean value of both hands was used for analysis.

### Lifestyle and health assessment

Information on lifestyle behaviors, including energy intake and physical activity, was collected using self-administered questionnaires and dietary records. Daily energy intake was calculated by a registered dietitian based on a 7-day food record completed by each participant. Total physical activity was assessed using the short-form Japanese version of the International Physical Activity Questionnaire (IPAQ) [16]. According to the IPAQ scoring protocol, physical activity was expressed as metabolic equivalent minutes per week (MET-min/week), calculated from walking, moderate-intensity, and vigorous-intensity activities (3.3, 4.0, and 8.0 METs, respectively). Low physical activity was defined as <600 MET-min/week [17]. Menstrual history, including dysmenorrhea, and dieting experience were also assessed. Anorexia nervosa (AN) was screened using the Eating Attitudes Test-26 (EAT-26), with a total score ≥20 considered indicative of possible AN [18]. Depressive symptoms were evaluated using the Patient Health Questionnaire-2 (PHQ-2), which assesses depressed mood and anhedonia over the preceding two weeks; a total score ≥3 was considered indicative of possible depression [19].

### Laboratory measurements

Blood samples were collected to assess renal and liver function, lipid profile, serum UA levels, and glycated hemoglobin (HbA1c).

### Muscle mass and strength classification

Because no diagnostic criteria for low muscle mass and low muscle strength have been specifically established for young women, participants were classified according to the AWGS 2019 criteria [9], which provide established diagnostic cut-off values for skeletal muscle mass and handgrip strength. Low muscle mass was defined as SMI < 5.7 kg/m² measured by bioelectrical impedance analysis (BIA), and low muscle strength was defined as handgrip strength <18 kg. Participants were initially categorized into four groups based on the presence or absence of low muscle mass and low muscle strength. However, no participant exhibited low muscle strength in the absence of low muscle mass. Therefore, participants were ultimately classified into three groups: normal muscle status, low muscle mass only and low muscle mass with reduced handgrip strength. As a sensitivity analysis, low muscle mass was also defined as an SMI < 5.0 kg/m², according to a validation study of 154 Japanese women aged 18–40 years using the InBody 770. In that study, SMI was calculated as appendicular lean mass divided by height squared (ALM/Ht²), and a cutoff value of 5.0 kg/m² was proposed for InBody-derived SMI measurements [20].

### Ethics approval

This study was approved by the Ethics Committee of Shimane University, Japan (Approval No. 20220422−1). Written informed consent was obtained from all participants prior to study participation. The study was conducted in accordance with the principles of the Declaration of Helsinki.

### Statistical analysis

Continuous variables were presented as mean ± standard deviation (SD) when normally distributed and as median (interquartile range [IQR]) when non-normally distributed. Categorical variables were presented as counts and percentages.

Serum UA levels were compared among the three muscle status groups (normal muscle status, low muscle mass only, and low muscle mass with reduced handgrip strength) using one-way analysis of variance (ANOVA), followed by Tukey’s post hoc test for pairwise comparisons.

Associations between serum UA and muscle-related parameters, including handgrip strength and SMI, were examined using Pearson correlation analysis. Multivariable linear regression analyses were performed with handgrip strength as the dependent variable and serum UA as the primary independent variable. Three models were constructed. Model 1 was adjusted for age. Model 2 was additionally adjusted for SMI and estimated glomerular filtration rate (eGFR). Model 3 was further adjusted for daily energy intake and physical activity. Regression coefficients (B) with 95% confidence intervals (CIs) were calculated. Multicollinearity was assessed using variance inflation factors (VIFs). All statistical analyses were performed using StatFlex version 7 (Artec Co., Ltd., Osaka, Japan), and a two-sided p value <0.05 was considered statistically significant.

## Results

### Baseline characteristics

There were no significant differences in age, BMI, or handgrip strength between the included (n = 73) and excluded participants (n = 20).

Among the 73 participants, the mean age was 19.2 ± 1.4 years, and the mean BMI was 17.2 ± 0.9 kg/m². Mean SMI and handgrip strength were 5.1 ± 0.5 kg/m² and 21.9 ± 3.9 kg, respectively. Mean daily energy intake was 1,547 ± 315 kcal/day, and the median physical activity level was 693 (396–1,188) MET-min/week, with 38.4% of participants classified as low physical activity (<600 MET-min/week). No participants screened positive for AN. Laboratory measurements showed a mean serum UA level of 3.9 ± 0.7 mg/dL, and 11.0% of participants had a UA level ≤3.0 mg/dL. Mean serum creatinine and eGFR were 0.5 ± 0.1 mg/dL and 122 ± 21 mL/min/1.73 m², respectively (Table 1).

**Table 1:** Baseline characteristics of the participants.

	Total (N = 73)
Age (years)	19.2 ± 1.4
Height (cm)	157.5 ± 5.5
Weight (kg)	42.7 ± 3.7
BMI (kg/m²)	17.2 ± 0.9
Systolic BP (mmHg)	116 ± 14
Heart rate (bpm)	88 ± 17
SMM (kg)	17.6 ± 2.1
SMI (kg/m²)	5.1 ± 0.5
Fat percentage (%)	22.3 ± 4.6
Handgrip strength (kg)	21.9 ± 3.9
Daily energy intake (kcal/day)	1,547 ± 315
Physical activity (MET-min/week)	693(396−1,188)
Low physical activity (<600 MET-min/week) (%)	38.4
Dysmenorrhea (%)	45.1
Dieting experience (%)	15.0
Screened positive for anorexia nervosa (%)	0
Depressive symptoms (%)	8.2
Laboratory measurements	
UA (mg/dL)	3.9 ± 0.7
UA ≤ 3.0 mg/dL (%)	11.0
BUN (mg/dL)	11.4 ± 3.0
Creatinine (mg/dL)	0.5 ± 0.1
eGFR (mL/min/1.73 m²)	122 ± 21
AST (U/L)	21 (20-24)
ALT (U/L)	11 (9-15)
γ-GTP (U/L)	10 (7-13)
TG (mg/dL)	70 (55-95)
T-C (mg/dL)	156 (146-172)
LDL C(mg/dL)	81 (73-93)
HDL C(mg/dL)	66 (58-72)
Total protein (g/dL)	7.4 ± 0.2
Albumin (g/dL)	4.6 ± 0.1
Hemoglobin A1c (%)	5.2 ± 0.2

BMI: body mass index; BP: blood pressure; SMM: skeletal muscle mass; SMI: skeletal muscle index. BUN: Blood urea nitrogen, TG: Triglycerides, T-C: Total cholesterol, LDL-C: Low-density lipoprotein cholesterol, HDL-C: High-density lipoprotein cholesterol, UA: uric acid.

Using the AWGS 2019 criteria (SMI < 5.7 kg/m²), 63 of 73 participants (86.3%) were classified as having low muscle mass. In a sensitivity analysis using an alternative cutoff of SMI < 5.0 kg/m² proposed in a validation study of young Japanese women [20], 24 participants (32.9%) were classified as having low muscle mass, resulting in reclassification of 39 participants (53.4%).

### Serum UA levels according to muscle mass and strength status

Serum UA levels differed significantly among the three muscle status groups (one-way ANOVA, p = 0.026; Table 2). Serum UA levels were lowest in participants with both low muscle mass and reduced handgrip strength. Post hoc analysis showed that this group had significantly lower serum UA levels than the low muscle mass only group (3.4 ± 0.7 vs. 4.1 ± 0.6 mg/dL, p = 0.020). No significant differences were observed in the other pairwise comparisons.

**Table 2:** Serum UA levels according to muscle mass and handgrip strength status.

Group	n	Serum UA (mg/dL)	one-way ANOVA p value
Normal	10	3.9 ± 0.8	0.026
Low muscle mass only	52	4.1 ± 0.6	
Low muscle mass + low handgrip strength	11	3.4 ± 0.7†	

Data are presented as mean ± SD. Overall comparisons were performed using one-way ANOVA followed by Tukey’s post hoc test.

† p = 0.020 versus the low muscle mass only group (Tukey’s post hoc test).

### Associations between serum UA and muscle-related parameters

**Fig 1:**
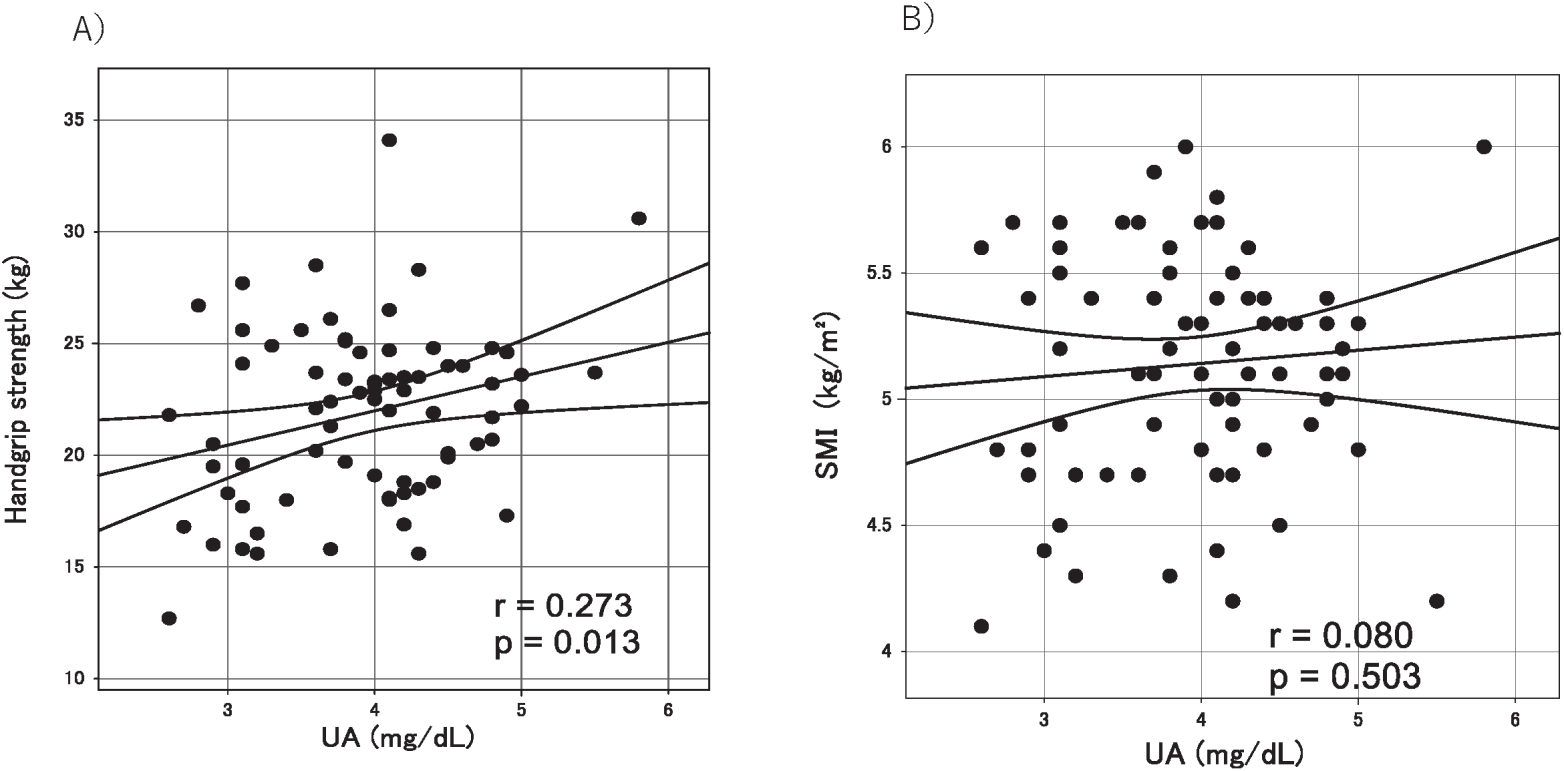
Relationship between serum uric acid and muscle-related parameters. Scatter plots show the associations between serum UA and (A) handgrip strength and (B) SMI in underweight young women. The solid line represents the fitted linear regression line, and the outer lines indicate the 95% confidence bands.

Serum UA was positively correlated with handgrip strength (r = 0.273, p = 0.013). No significant correlation was observed between serum UA and SMI (r = 0.080, p = 0.503) (Fig 1).

### Multivariable linear regression analysis

In the age-adjusted model (Model 1), serum UA was positively associated with handgrip strength (B = 1.605, 95% CI 0.316–2.893, p = 0.015). After further adjustment for SMI and eGFR (Model 2), the association remained significant (B = 1.186, 95% CI 0.061–2.311, p = 0.039). This association persisted after additional adjustment for daily energy intake and physical activity (Model 3; B = 1.245, 95% CI 0.060–2.431, p = 0.040). SMI was independently associated with handgrip strength (B = 4.605, 95% CI 2.723–6.487, p < 0.001), whereas age, eGFR, daily energy intake, and physical activity were not significantly associated with handgrip strength (Table 3). All VIFs in Model 3 were below 1.3, indicating no evidence of problematic multicollinearity.

**Table 3:** Multivariable linear regression analysis of factors associated with handgrip strength.

Variable	Model 1 B (95% CI)	p	Model 2 B (95% CI)	p	Model 3 B (95% CI)	p	VIF
Age (years)	−0.297 (−0.944, 0.349)	0.362	−0.361 (−0.883, 0.163)	0.174	−0.309 (−0.869, 0.250)	0.273	1.07
UA (mg/dL)	1.605 (0.316, 2.893)	0.015	1.186 (0.061, 2.311)	0.039	1.245 (0.060, 2.431)	0.040	1.21
SMI (kg/m^2^)	—	—	4.797 (3.179, 6.416)	<0.001	4.605 (2.723, 6.487)	<0.001	1.25
eGFR (mL/min/1.73 m²)	—	—	−0.016 (−0.053, 0.022)	0.407	−0.012 (−0.053, 0.029)	0.567	1.15
Daily energy intake (kcal/day)	—	—	—	—	0.002 (−0.001, 0.004)	0.211	1.24
Physical activity (MET-min/week)	—	—	—	—	0.0001 (−0.0006, 0.0008)	0.792	1.20

Model 1: Adjusted for age.

Model 2: Additionally adjusted for SMI and eGFR

Model 3: Additionally adjusted for daily energy intake and physical activity.

UA, uric acid; SMI, skeletal muscle index; eGFR, estimated glomerular filtration rate; CI, confidence interval; VIF, variance inflation factor.

## Discussion

In this exploratory study of underweight young women, serum UA levels were associated with handgrip strength but not with skeletal muscle mass. In multivariable linear regression analyses, serum UA remained independently associated with handgrip strength after adjustment for age, SMI, eGFR, daily energy intake, and physical activity. These findings suggest that serum UA may be more closely associated with muscle function than with muscle mass in underweight young women.

Sarcopenia, characterized by declines in muscle strength and muscle mass, is a widely used construct for evaluating muscle health in clinical research [6,10]. Previous studies examining the association between UA and sarcopenia have reported positive, negative, and nonlinear relationships. In a four-year cohort study of 5,086 Chinese adults aged ≥45 years, men in the highest UA quartile (≥5.7 mg/dL) had a significantly lower risk of incident sarcopenia than those in the lowest quartile (HR 0.57, 95% CI 0.41–0.80), whereas no significant association was observed in women [1]. Higher UA levels within the normal range have also been associated with greater muscle mass and handgrip strength in observational studies [2,3]. In contrast, NHANES data suggested a U-shaped relationship between UA and muscle health. Participants with UA ≥ 6.3 mg/dL had a higher prevalence of low appendicular lean mass relative to BMI than those in the lowest UA group (10.5% vs. 5.3%), with the lowest risk observed at approximately UA 4.5–5.5 mg/dL [4]. Furthermore, a recent bi-directional Mendelian randomization study found no causal effect of serum urate on handgrip strength, lean mass, or gait speed [5]. Taken together, these findings suggest that serum UA may be associated with muscle health, although it may primarily reflect underlying metabolic or oxidative processes rather than directly influence skeletal muscle.

The inconsistent findings across studies may be partly explained by differences in age, sex, and nutritional status among study populations. Previous studies have suggested that moderately higher UA levels are associated with better muscle health in middle-aged and older adults, whereas the relationship appears less consistent in younger populations [1,2,4]. In addition, estrogen enhances renal UA excretion, resulting in lower UA levels in premenopausal women, which may partly contribute to sex-related differences in the association between UA and muscle-related outcomes.

UA is the end product of purine metabolism and is closely linked to cellular energy turnover. Under physiological conditions, efficient adenosine triphosphate (ATP) resynthesis limits purine degradation and maintains UA production within a normal range. Conversely, energy deficiency may alter ATP metabolism and purine turnover, thereby influencing serum UA levels [21]. Miller et al. demonstrated that fasting and glucocorticoid/FoxO3 signaling promote purine degradation and release from skeletal muscle, resulting in increased UA production through xanthine oxidoreductase-dependent pathways [22]. However, prolonged undernutrition may ultimately reduce the availability of substrates required for purine metabolism. In the present study, participants had a mean energy intake of approximately 1,550 kcal/day, which was below the estimated energy requirement even for Japanese women aged 18–29 years with a low physical activity level (1,700 kcal/day) [23]. Furthermore, 38.4% of participants had low physical activity and 45.1% reported dysmenorrhea, indicating that this cohort exhibited several characteristics commonly observed in underweight young women. These findings suggest that many participants may have had suboptimal nutritional and physiological status. Lower serum UA levels may therefore reflect altered nutritional and metabolic conditions in this population.

In addition, serum UA may be associated with muscle function through its antioxidant properties. UA is one of the major endogenous antioxidants in humans [21]. Lower serum UA levels may reduce antioxidant capacity and increase oxidative stress. Oxidative stress has been reported to impair muscle protein homeostasis and contractile function, potentially contributing to reduced muscle strength [24]. These mechanisms may partly explain why serum UA was more closely associated with muscle strength than muscle mass in the present study. However, these proposed mechanisms should be interpreted with caution because this was a cross-sectional study and oxidative stress and metabolic biomarkers were not assessed.

Another important consideration is the applicability of the diagnostic criteria used in this study. The AWGS 2019 criteria were originally developed for older Asian adults and may not be fully applicable to younger populations. A sensitivity analysis using an alternative SMI cutoff of <5.0 kg/m² for young Japanese women [20] reduced the prevalence of low muscle mass from 86.3% to 32.9% in our cohort. This marked difference highlights the need for age-specific diagnostic criteria for young women. Nevertheless, we defined low muscle mass and low muscle strength according to the AWGS 2019 criteria because they represent the current consensus diagnostic criteria for Asian populations and facilitate comparison with previous studies. For handgrip strength, published age-specific normative data for healthy women aged 20–39 years reported 5th percentile handgrip strength values of 18–20 kg [7], which closely correspond to the AWGS cutoff (<18 kg). Therefore, the classification of low muscle strength is unlikely to differ substantially. Our primary regression analyses evaluated SMI and handgrip strength as continuous variables; therefore, the overall conclusions regarding the association between serum UA and muscle function were unlikely to be materially influenced by the choice of categorical cutoff.

This study has several limitations. First, its cross-sectional design precludes causal inference, and the relatively small sample size, particularly the small subgroup of participants with both low muscle mass and low muscle strength, may have limited statistical power. However, this study focused on a highly specific population of underweight young women who underwent comprehensive assessments, which limited the number of eligible participants while reducing potential confounding factors. Although body fat percentage may be a useful indicator of nutritional status, it was not included in the final multivariable model because it is related to skeletal muscle mass. Given the relatively large number of covariates compared with the sample size, further inclusion of variables might have increased the risk of over-adjustment and overfitting. In addition, because participants were recruited from a single university, the findings may not be generalizable to other populations, including young men or young adults with normal body weight. Second, muscle mass was assessed using a single BIA device rather than dual-energy X-ray absorptiometry (DXA). Because BIA is influenced by hydration status and is less accurate than DXA, measurement error cannot be excluded. Third, renal function was assessed using creatinine-based eGFR rather than cystatin C-based eGFR. Because creatinine-based eGFR is influenced by muscle mass, residual confounding related to renal function cannot be excluded [25,26]. Fourth, detailed nutritional factors and female sex hormone levels, both of which may influence muscle health and UA metabolism, were not assessed. In addition, serum UA levels may vary across the menstrual cycle because of hormonal fluctuations [27], and underweight women may experience menstrual irregularities associated with altered hormone levels. Finally, oxidative stress and metabolic biomarkers were not assessed; therefore, the biological mechanisms underlying the observed association remain speculative. Future longitudinal studies incorporating comprehensive nutritional, hormonal, and metabolic assessments are warranted.

Despite these limitations, little is known about the association between serum UA and muscle-related parameters in underweight young women. Therefore, the present findings provide preliminary evidence in this understudied population.

In conclusion, serum UA was associated with handgrip strength but not with skeletal muscle mass in underweight young women. These findings suggest that serum UA may be more closely related to muscle function than to muscle mass in this population. Further prospective studies are warranted to clarify the underlying mechanisms and clinical implications of this association.
