# Supplementary material for: Association between serum uric acid and muscle function in underweight young women: An exploratory cross-sectional study
Source: PLoS One. 2026 Aug 14;21(8):e0355968. doi: 10.1371/journal.pone.0355968 (PMC13475819; doi:10.1371/journal.pone.0355968)
Supplement: Supplementary file 2 [file pone.0355968.s002.docx]

**Dear Reviewers**

We sincerely thank the reviewers for the positive evaluation of our revised manuscript and for their helpful comments. We have addressed the remaining comments as follows.

**Reviewer 1**

**Comment 2. Confounding adjustment**
The inclusion of daily energy intake and physical activity in the multivariable model strengthens the analysis.
However, body fat percentage was measured and could also reflect nutritional status. Although I understand the concern regarding over-adjustment given the limited sample size, a brief explanation in the Discussion describing why body fat percentage was not included in the final model would improve transparency.

**Response:**
Thank you for this valuable suggestion. We have added an explanation in the Discussion stating that although body fat percentage may be a useful indicator of nutritional status, it was not included in the final multivariable model because it is related to skeletal muscle mass, and further inclusion of variables was not considered appropriate given the sample size. The manuscript has been revised accordingly (Discussion, page 19, lines 293–296).

**Comment 3. Sample size**
The authors appropriately acknowledge the limited sample size and report VIF values.
One additional sentence discussing the possibility of overfitting because of the relatively large number of covariates compared with the sample size would further strengthen the limitations section.

**Response:**
Thank you for this helpful comment. We have revised the Limitations section to acknowledge that, given the relatively large number of covariates compared with the sample size, further inclusion of variables might have increased the risk of over-adjustment and overfitting. The manuscript has been revised accordingly (Discussion, page 19, lines 295–296).

**Minor editorial comments:** There are still a few minor language issues. For example,
"muscle quantity. In the present study..." should read "muscle quantity. In the present study..."Similarly, several spaces around references (e.g., "[19][21]") should be standardized according to journal style. A final careful English proofreading is recommended before publication.

**Response:**
Thank you for this helpful comment. We have carefully proofread the manuscript and corrected the remaining minor language and formatting issues to conform to the journal style.
