# Supplementary material for: Association between serum uric acid and muscle function in underweight young women: An exploratory cross-sectional study
Source: PLoS One. 2026 Aug 14;21(8):e0355968. doi: 10.1371/journal.pone.0355968 (PMC13475819; doi:10.1371/journal.pone.0355968)
Supplement: Supplementary file 3 [file pone.0355968.s001.docx]

Dear Editor and Reviewers,

Thank you for your careful review and valuable comments on our manuscript. We have revised the manuscript accordingly, and our point-by-point responses are provided below.

**Reviewer #1**

**Comment 1. Regression model specification should be reconsidered.**
The authors modeled serum UA as the dependent variable, while the manuscript consistently discusses UA as a predictor of muscle function. This creates conceptual inconsistency between the statistical analysis and the biological interpretation.
Although mathematically acceptable in cross-sectional analyses, readers naturally interpret the manuscript as asking whether UA predicts handgrip strength rather than vice versa.
The authors should justify this modeling strategy more explicitly. Alternatively, regression analyses using handgrip strength as the dependent variable and UA as an explanatory variable would align better with the study objective and improve interpretability.
**Response**

Thank you for this insightful comment. We revised the multivariable linear regression analyses using handgrip strength as the dependent variable and serum UA as the primary independent variable. Three sequential models were constructed with adjustment for age, SMI, eGFR, daily energy intake, and physical activity. Serum UA remained independently associated with handgrip strength after adjustment for these variables. The manuscript has been revised accordingly (Methods, page 9–10, lines 146–155; Results, page 14, lines 199–207; Table 3, pages 14; Discussion, page 15, lines 216–218).

**Comment 2. Important confounding variables were measured but not included in multivariable analyses.**
The study collected valuable information on
• daily energy intake,
• physical activity,
• body fat percentage,
• depressive symptoms,
• dieting history,
Yet none of these variables were incorporated into the regression models. Because UA is strongly influenced by nutritional status and energy metabolism, the observed association between UA and grip strength may simply reflect overall nutritional condition. At minimum, the authors should perform sensitivity analyses adjusting for energy intake and physical activity or explain why these variables were omitted.
**Response**

Thank you for this valuable comment. As described in our response to Comment 1, we performed additional multivariable linear regression analyses adjusting for daily energy intake and physical activity. Serum UA remained independently associated with handgrip strength after adjustment for these variables.

**Comment 3. Sample size and model stability**
The study includes only 73 participants. Considering the number of explanatory variables and subgroup comparisons, the regression model may be underpowered. The authors should report whether any assessment of model stability or multicollinearity (e.g., variance inflation factor) was performed.
**Response**

Thank you for this valuable comment. We evaluated multicollinearity using variance inflation factors (VIFs) to assess model stability. All VIFs were below 1.3, indicating no evidence of problematic multicollinearity. We also clarified in the Discussion that the relatively small sample size is a limitation of the present study. The manuscript has been revised accordingly (Methods, page 10 lines 152–153; Results, page 14, lines 206–207; Table 3, pages 14).

**Comment 4. Interpretation should be more cautious.**
The discussion repeatedly suggests that UA may reflect muscle function through antioxidant or energy metabolic mechanisms. However, this study is cross-sectional, and no oxidative stress markers or metabolic biomarkers were measured. The mechanistic discussion is interesting but currently somewhat speculative and should be toned down.
**Response**

Thank you for this valuable comment. We revised the Discussion to present the proposed mechanisms more cautiously and to emphasize that they remain speculative because of the cross-sectional design and the absence of direct measurements of oxidative stress and metabolic biomarkers. The manuscript has been revised accordingly (Discussion, page 18, lines 266–270, page 20, lines 305–308).

**Comment 5. Use of AWGS criteria in young women**
The authors appropriately acknowledge that AWGS criteria were developed for older adults.
However, this limitation deserves more discussion. Specifically, readers would benefit from understanding how many participants would have been classified differently using age-specific normative values rather than sarcopenia cutoffs intended for elderly populations.
**Response**

Thank you for this valuable comment. We further discussed the applicability of the AWGS 2019 criteria in young women. We also added a reference proposing an alternative SMI cutoff for young Japanese women (Reference 20) and performed a sensitivity analysis. Although the prevalence of low muscle mass differed, the main conclusions remained unchanged. The manuscript has been revised accordingly (Methods, page 8, lines 127–131; Results, page 11, lines 168–172; Discussion, page 18–19, lines 271–286; Reference 20).

**Minor Comments
Comment 1. Clarify participant selection.**
Twenty participants (93 enrolled vs. 73 analyzed) were excluded because of incomplete data.
Please specify which variables were missing and whether excluded participants differed from included participants.

**Response**
Thank you for this helpful comment. We clarified in the Methods section that 20 participants were excluded because blood test data were unavailable. We also compared the included and excluded participants and found no significant differences in age, BMI, or handgrip strength. The manuscript has been revised accordingly (Methods, page 6, lines 87–88; Results, page 10, lines 158–159).

**Comment 2. Data availability statement**
The statement indicates that data are available upon reasonable request. Since PLOS ONE strongly encourages unrestricted public data sharing, the authors should explain whether ethical restrictions prevent public deposition or consider depositing anonymized data in an appropriate repository.

**Response:**
Thank you for this important comment. We revised the Data Availability Statement in the submission system to clarify that the data cannot be deposited in a public repository because participants did not provide consent for public data sharing. We also specified that requests for access to de-identified data may be submitted to the Matsue Health Service Center, Shimane University, and reviewed in accordance with applicable ethical, legal, and institutional requirements.

**Comment 3. Figure 1**
Please include confidence bands around the regression lines. This would improve visualization of the uncertainty around the estimated association.
**Response:**
Thank you for this helpful comment. We added 95% confidence bands around the regression lines in Figure 1 to better illustrate the uncertainty of the estimated associations. The manuscript has been revised accordingly (Figure 1, page 13).

**Comment 4. Table 2**
Only one post hoc comparison reached statistical significance. Please report the overall ANOVA P-value in the table.

**Response:**
Thank you for this helpful comment. We added the overall ANOVA p-value to Table 2. The manuscript has been revised accordingly (Table 2, page 12-13).

**Comment 5. Language**
The manuscript is generally well written. Only minor English editing is suggested to improve readability, particularly in the discussion, where several paragraphs are relatively long.

**Response:**

Thank you for this helpful comment. We carefully revised the English throughout the manuscript to improve readability, particularly in the Discussion.

**Reviewer #2**

**Comment 1.**
Although lifestyle factors (energy intake, physical activity, menstrual abnormalities, psychological status) were collected, they were not included in the regression models. These variables strongly influence muscle strength and may confound the observed association between uric acid and handgrip strength.
**Response:**

Thank you for this valuable comment. We revised the multivariable linear regression analyses by additionally adjusting for daily energy intake and physical activity. Serum UA remained independently associated with handgrip strength after these adjustments. The manuscript has been revised accordingly (Methods, page 9–10, lines 146–155; Results, page 14, lines 199–207; Table 3, pages 14; Discussion, page 15, lines 216–218).

**Comment 2.**
The AWGS 2019 cutoffs were developed for older adults. Their use in young women requires clearer justification, and the limitations of applying geriatric thresholds to a young population should be explicitly acknowledged.

**Response:**

Thank you for this valuable comment. We further discussed the applicability of the AWGS 2019 criteria in young women. We also added a reference proposing an alternative SMI cutoff for young Japanese women (Reference 20) and performed a sensitivity analysis. Although the prevalence of low muscle mass differed, the main conclusions remained unchanged. The manuscript has been revised accordingly (Methods, page 8, lines 127–131; Results, page 11, lines 168–172; Discussion, page 18–19, lines 271–286; Reference 20).

**Comment 3.**
Muscle mass was assessed using a single BIA device, which is sensitive to hydration status and less accurate than DXA. This methodological limitation should be more clearly discussed.
**Response:**

Thank you for this valuable comment. We added the methodological limitations of BIA to the Limitations section of the Discussion. The manuscript has been revised accordingly (Discussion, page 19, lines 295–297).

**Comment 4.**
The study design precludes causal inference. It remains unclear whether uric acid influences muscle strength, reflects underlying nutritional/metabolic status, or is affected by other unmeasured factors.

**Response:**

Thank you for this valuable comment. We revised the Discussion to emphasize that the cross-sectional design precludes causal inference and that the observed association may reflect underlying nutritional or metabolic status or be influenced by other unmeasured factors. The manuscript has been revised accordingly (Discussion, page 18, lines 266–270; page 20, lines 305–308).

**Comment 5.**
The discussion relies heavily on previous literature and does not sufficiently interpret the findings in the specific context of underweight young women. More emphasis on unique characteristics of this population (low energy availability, low protein intake, menstrual irregularities) would strengthen the manuscript.
**Response:**

Thank you for this valuable comment. We revised the Discussion to place greater emphasis on interpreting our findings in the specific context of underweight young women and streamlined the descriptions of previous studies. To provide a more detailed characterization of this study population, we additionally calculated the prevalence of low physical activity (38.4%) and compared the participants’ mean energy intake with the estimated energy requirement (Reference 23). We also incorporated the previously reported prevalence of menstrual irregularities (45.1%) into the interpretation of our findings. In addition, we clarified that detailed analyses of nutritional factors, including protein intake, were not performed in this study. These population characteristics were discussed as important contextual factors for interpreting the observed association between serum uric acid and muscle function. The manuscript has been revised accordingly (Methods, page 7, lines 106–107; Results, page 10, lines 163–164; Table 1, page 11; Discussion, page 17, lines 253–261; Reference 23).

**Comment 6.**
The potential mechanisms linking uric acid to muscle function—antioxidant activity, energy metabolism, nutritional status—should be elaborated to enhance biological plausibility.

**Response:**
Thank you for this valuable comment. We expanded the Discussion to further elaborate the biological plausibility of our findings. Specifically, we added a discussion on the potential roles of uric acid in antioxidant defense and nutritional/metabolic status, including altered energy metabolism associated with undernutrition. We also noted that these mechanisms remain speculative because of the cross-sectional design and the lack of direct measurements of oxidative stress and metabolic biomarkers. The manuscript has been revised accordingly (Discussion, page 17-18, lines 253–270).

**Comment** 7.
The overall sample size is modest, and the subgroup with low muscle mass and low strength is particularly small. This should be acknowledged as a limitation affecting statistical power.

**Response:**
Thank you for this valuable comment. We clarified in the Limitations section that the relatively small overall sample size and the small subgroup of participants with both low muscle mass and low muscle strength may have limited the statistical power of the analyses. The manuscript has been revised accordingly (Discussion, page 19, lines 287–290).

**Others;**
Participants were drawn from a single university and represent a specific demographic group. External validity to other young adults or to males is limited.

**Response:**
Thank you for this helpful comment. We revised the Limitations section to clarify that participants were recruited from a single university and included only underweight young women, which may limit the generalizability of the findings to other populations, including young men and young adults with normal body weight. The manuscript has been revised accordingly (Discussion, page 19, lines 292–295).
