# Supplementary material for: Association between serum uric acid and muscle function in underweight young women: An exploratory cross-sectional study
Source: PLoS One. 2026 Aug 14;21(8):e0355968. doi: 10.1371/journal.pone.0355968 (PMC13475819; doi:10.1371/journal.pone.0355968)
Supplement: Peer review history

# Peer review: Decision Letter 0

**DOI:** 10.1371/journal.pone.0355968.r001

**Authors:** Tomohiro Yasuda

17 Jul 2026

Dear Dr. Sugihara,

Thank you for submitting your manuscript to PLOS ONE. After careful consideration, we feel that it has merit but does not fully meet PLOS ONE’s publication criteria as it currently stands. Therefore, we invite you to submit a revised version of the manuscript that addresses the points raised during the review process.

Please submit your revised manuscript by Sep 15 2026 11:59PM. If you will need more time than this to complete your revisions, please reply to this message or contact the journal office at plosone@plos.org. When you're ready to submit your revision, log on to https://www.editorialmanager.com/pone/ and select the 'Submissions Needing Revision' folder to locate your manuscript file.

- A letter that responds to each point raised by the academic editor and reviewer(s). You should upload this letter as a separate file labeled 'Response to Reviewers'.
- A marked-up copy of your manuscript that highlights changes made to the original version. You should upload this as a separate file labeled 'Revised Manuscript with Track Changes'.
- An unmarked version of your revised paper without tracked changes. You should upload this as a separate file labeled 'Manuscript'.

If you would like to make changes to your financial disclosure, please include your updated statement in your cover letter. Guidelines for resubmitting your figure files are available below the reviewer comments at the end of this letter.

If applicable, we recommend that you deposit your laboratory protocols in protocols.io to enhance the reproducibility of your results. Protocols.io assigns your protocol its own identifier (DOI) so that it can be cited independently in the future. For instructions see: https://journals.plos.org/plosone/s/submission-guidelines#loc-laboratory-protocols. Additionally, PLOS ONE offers an option for publishing peer-reviewed Lab Protocol articles, which describe protocols hosted on protocols.io. Read more information on sharing protocols at https://plos.org/protocols?utm_medium=editorial-email&utm_source=authorletters&utm_campaign=protocols.

As the corresponding author, your ORCID iD is verified in the submission system and will appear in the published article. PLOS supports the use of ORCID, and we encourage all coauthors to register for an ORCID iD and use it as well. Please encourage your coauthors to verify their ORCID iD within the submission system before final acceptance, as unverified ORCID iDs will not appear in the published article. *Only*  the individual author can complete the verification step; PLOS staff *cannot*  verify ORCID iDs on behalf of authors.

We look forward to receiving your revised manuscript.

Kind regards,

Tomohiro Yasuda, Ph.D.

Academic Editor

PLOS One

Journal Requirements:

If the reviewer comments include a recommendation to cite specific previously published works, please review and evaluate these publications to determine whether they are relevant and should be cited. There is no requirement to cite these works unless the editor has indicated otherwise.

[Note: HTML markup is below. Please do not edit.]

Reviewers' comments:

Reviewer's Responses to Questions

**Comments to the Author**

1. Is the manuscript technically sound, and do the data support the conclusions?

Reviewer #1: Partly

Reviewer #2: Partly

**********

2. Has the statistical analysis been performed appropriately and rigorously?

Reviewer #1: Yes

Reviewer #2: N/A

**********

3. Have the authors made all data underlying the findings in their manuscript fully available?

The PLOS Data policy

Reviewer #1: No

Reviewer #2: Yes

**********

4. Is the manuscript presented in an intelligible fashion and written in standard English?

Reviewer #1: Yes

Reviewer #2: Yes

**********

Reviewer #1: Overall Comments

This manuscript investigates the association between serum uric acid (UA) levels and muscle function in underweight young women, a relatively understudied population. The topic is clinically relevant because underweight young women may represent a unique group at risk of impaired muscle health, and the study addresses an important gap in the literature. The manuscript is generally well written, the rationale is clear, and the results are presented in a logical manner.

However, the study has several methodological and statistical limitations that should be addressed before publication. In particular, the interpretation of the regression analysis, handling of potential confounders, and discussion of the biological mechanisms require further clarification. Therefore, I recommend major revision.

Major Comments

1. Regression model specification should be reconsidered.

The authors modeled serum UA as the dependent variable, while the manuscript consistently discusses UA as a predictor of muscle function. This creates conceptual inconsistency between the statistical analysis and the biological interpretation.

Although mathematically acceptable in cross-sectional analyses, readers naturally interpret the manuscript as asking whether UA predicts handgrip strength rather than vice versa.

The authors should justify this modeling strategy more explicitly. Alternatively, regression analyses using handgrip strength as the dependent variable and UA as an explanatory variable would align better with the study objective and improve interpretability.

2. Important confounding variables were measured but not included in multivariable analyses.

The study collected valuable information on

• daily energy intake,

• physical activity,

• body fat percentage,

• depressive symptoms,

• dieting history,

Yet none of these variables were incorporated into the regression models.

Because UA is strongly influenced by nutritional status and energy metabolism, the observed association between UA and grip strength may simply reflect overall nutritional condition.

At minimum, the authors should perform sensitivity analyses adjusting for energy intake and physical activity or explain why these variables were omitted.

3. Sample size and model stability

The study includes only 73 participants.

Considering the number of explanatory variables and subgroup comparisons, the regression model may be underpowered.

The authors should report whether any assessment of model stability or multicollinearity (e.g., variance inflation factor) was performed.

4. Interpretation should be more cautious.

The discussion repeatedly suggests that UA may reflect muscle function through antioxidant or energy metabolic mechanisms.

However, this study is cross-sectional, and no oxidative stress markers or metabolic biomarkers were measured.

The mechanistic discussion is interesting but currently somewhat speculative and should be toned down.

5. Use of AWGS criteria in young women

The authors appropriately acknowledge that AWGS criteria were developed for older adults.

However, this limitation deserves more discussion.

Specifically, readers would benefit from understanding how many participants would have been classified differently using age-specific normative values rather than sarcopenia cutoffs intended for elderly populations.

Minor Comments

1. Clarify participant selection.

Twenty participants (93 enrolled vs. 73 analyzed) were excluded because of incomplete data.

Please specify which variables were missing and whether excluded participants differed from included participants.

2. Data availability statement

The statement indicates that data are available upon reasonable request.

Since PLOS ONE strongly encourages unrestricted public data sharing, the authors should explain whether ethical restrictions prevent public deposition or consider depositing anonymized data in an appropriate repository.

3. Figure 1

Please include confidence bands around the regression lines.

This would improve visualization of the uncertainty around the estimated association.

4. Table 2

Only one post hoc comparison reached statistical significance.

Please report the overall ANOVA P-value in the table.

5. Language

The manuscript is generally well written.

Only minor English editing is suggested to improve readability, particularly in the discussion, where several paragraphs are relatively long.

Reviewer #2: Thank you very much for submitting this manuscript for review. This manuscript is interesting for readers. However, there are few comments for this.

NO1;

Although lifestyle factors (energy intake, physical activity, menstrual abnormalities, psychological status) were collected, they were not included in the regression models. These variables strongly influence muscle strength and may confound the observed association between uric acid and handgrip strength.

NO2;

The AWGS 2019 cutoffs were developed for older adults. Their use in young women requires clearer justification, and the limitations of applying geriatric thresholds to a young population should be explicitly acknowledged.

NO3;

Muscle mass was assessed using a single BIA device, which is sensitive to hydration status and less accurate than DXA. This methodological limitation should be more clearly discussed.

NO4;

The study design precludes causal inference. It remains unclear whether uric acid influences muscle strength, reflects underlying nutritional/metabolic status, or is affected by other unmeasured factors.

NO5;

The discussion relies heavily on previous literature and does not sufficiently interpret the findings in the specific context of underweight young women. More emphasis on unique characteristics of this population (low energy availability, low protein intake, menstrual irregularities) would strengthen the manuscript.

NO6;

The potential mechanisms linking uric acid to muscle function—antioxidant activity, energy metabolism, nutritional status—should be elaborated to enhance biological plausibility.

NO7;

The overall sample size is modest, and the subgroup with low muscle mass and low strength is particularly small. This should be acknowledged as a limitation affecting statistical power.

Others;

Participants were drawn from a single university and represent a specific demographic group. External validity to other young adults or to males is limited.

**********

what does this mean?). If published, this will include your full peer review and any attached files.

If you choose “no”, your identity will remain anonymous but your review may still be made public.

**Do you want your identity to be public for this peer review?**  For information about this choice, including consent withdrawal, please see our Privacy Policy

Reviewer #1: No

Reviewer #2: No

**********

[NOTE: If reviewer comments were submitted as an attachment file, they will be attached to this email and accessible via the submission site. Please log into your account, locate the manuscript record, and check for the action link "View Attachments". If this link does not appear, there are no attachment files.]

To ensure your figures meet our technical requirements, please review our figure guidelines: https://journals.plos.org/plosone/s/figures

You may also use PLOS’s free figure tool, NAAS, to help you prepare publication quality figures: https://journals.plos.org/plosone/s/figures#loc-tools-for-figure-preparation.

NAAS will assess whether your figures meet our technical requirements by comparing each figure against our figure specifications.

# Author comment: Author response to Decision Letter 1

**DOI:** 10.1371/journal.pone.0355968.r002

23 Jul 2026

Please see the attached "Response to Reviewers" document for our detailed point-by-point responses to all comments from the Academic Editor and reviewers.

Attachment Submitted filename: Response to Reviewers.docx

# Peer review: Decision Letter 1

**DOI:** 10.1371/journal.pone.0355968.r003

**Authors:** Tomohiro Yasuda

25 Jul 2026

Dear Dr.  Sugihara,

Thank you for submitting your manuscript to PLOS ONE. After careful consideration, we feel that it has merit but does not fully meet PLOS ONE’s publication criteria as it currently stands. Therefore, we invite you to submit a revised version of the manuscript that addresses the points raised during the review process.

Please submit your revised manuscript by Aug 24 2026 11:59PM If you will need more time than this to complete your revisions, please reply to this message or contact the journal office at plosone@plos.org. When you're ready to submit your revision, log on to https://www.editorialmanager.com/pone/ and select the 'Submissions Needing Revision' folder to locate your manuscript file.

- A letter that responds to each point raised by the academic editor and reviewer(s). You should upload this letter as a separate file labeled 'Response to Reviewers'.
- A marked-up copy of your manuscript that highlights changes made to the original version. You should upload this as a separate file labeled 'Revised Manuscript with Track Changes'.
- An unmarked version of your revised paper without tracked changes. You should upload this as a separate file labeled 'Manuscript'.

If you would like to make changes to your financial disclosure, please include your updated statement in your cover letter. Guidelines for resubmitting your figure files are available below the reviewer comments at the end of this letter.

If applicable, we recommend that you deposit your laboratory protocols in protocols.io to enhance the reproducibility of your results. Protocols.io assigns your protocol its own identifier (DOI) so that it can be cited independently in the future. For instructions see: https://journals.plos.org/plosone/s/submission-guidelines#loc-laboratory-protocols. Additionally, PLOS ONE offers an option for publishing peer-reviewed Lab Protocol articles, which describe protocols hosted on protocols.io. Read more information on sharing protocols at https://plos.org/protocols?utm_medium=editorial-email&utm_source=authorletters&utm_campaign=protocols.

As the corresponding author, your ORCID iD is verified in the submission system and will appear in the published article. PLOS supports the use of ORCID, and we encourage all coauthors to register for an ORCID iD and use it as well. Please encourage your coauthors to verify their ORCID iD within the submission system before final acceptance, as unverified ORCID iDs will not appear in the published article. *Only*  the individual author can complete the verification step; PLOS staff *cannot*  verify ORCID iDs on behalf of authors.

We look forward to receiving your revised manuscript.

Kind regards,

Tomohiro Yasuda, Ph.D.

Academic Editor

PLOS One

Journal Requirements:

1. If the reviewer comments include a recommendation to cite specific previously published works, please review and evaluate these publications to determine whether they are relevant and should be cited. There is no requirement to cite these works unless the editor has indicated otherwise.

2. Please review your reference list to ensure that it is complete and correct. If you have cited papers that have been retracted, please include the rationale for doing so in the manuscript text, or remove these references and replace them with relevant current references. Any changes to the reference list should be mentioned in the rebuttal letter that accompanies your revised manuscript. If you need to cite a retracted article, indicate the article’s retracted status in the References list and also include a citation and full reference for the retraction notice.

[Note: HTML markup is below. Please do not edit.]

Reviewer's Responses to Questions

**Comments to the Author**

Reviewer #1: All comments have been addressed

Reviewer #2: All comments have been addressed

**********

2. Is the manuscript technically sound, and do the data support the conclusions?

Reviewer #1: Yes

Reviewer #2: Yes

**********

3. Has the statistical analysis been performed appropriately and rigorously?

Reviewer #1: Yes

Reviewer #2: Yes

**********

4. Have the authors made all data underlying the findings in their manuscript fully available?

The PLOS Data policy

Reviewer #1: Yes

Reviewer #2: Yes

**********

5. Is the manuscript presented in an intelligible fashion and written in standard English?

Reviewer #1: Yes

Reviewer #2: Yes

**********

Reviewer #1: The authors have addressed nearly all of the previous comments satisfactorily. The manuscript has substantially improved, particularly with regard to the statistical modeling and interpretation of the findings. I appreciate the authors' careful revisions.

Only a few minor issues remain.

Comment 1. Regression model

The revised regression analysis now appropriately models handgrip strength as the dependent variable and serum UA as the explanatory variable. This revision greatly improves the consistency between the study objective, statistical analysis, and biological interpretation.

This concern has been fully addressed.

Comment 2. Confounding adjustment

The inclusion of daily energy intake and physical activity in the multivariable model strengthens the analysis.

However, body fat percentage was measured and could also reflect nutritional status. Although I understand the concern regarding over-adjustment given the limited sample size, a brief explanation in the Discussion describing why body fat percentage was not included in the final model would improve transparency.

Comment 3. Sample size

The authors appropriately acknowledge the limited sample size and report VIF values.

One additional sentence discussing the possibility of overfitting because of the relatively large number of covariates compared with the sample size would further strengthen the limitations section.

Comment 4. AWGS criteria

The newly added sensitivity analysis is useful and substantially improves the manuscript.

This concern has been adequately addressed.

Comment 5. Mechanistic interpretation

The discussion is now much more balanced.

The authors appropriately state that the proposed mechanisms remain speculative because oxidative stress markers were not measured.

This concern has been adequately addressed.

Minor editorial comments

There are still a few minor language issues.

For example,

"muscle quantity. In the present study..."

should read

"muscle quantity. In the present study..."

Similarly, several spaces around references (e.g., "[19][21]") should be standardized according to journal style.

A final careful English proofreading is recommended before publication.

Recommendation

Minor Revision

The major scientific concerns raised during the initial review have been adequately addressed. The remaining issues are minor and mainly concern clarification of model selection, acknowledgment of possible overfitting, and final editorial polishing.

Reviewer #2: Thank you very much for submitting this revised manuscript for review. There are no more comments for this.

**********

what does this mean?). If published, this will include your full peer review and any attached files.

If you choose “no”, your identity will remain anonymous but your review may still be made public.

**Do you want your identity to be public for this peer review?**  For information about this choice, including consent withdrawal, please see our Privacy Policy

Reviewer #1: No

Reviewer #2: No

**********

[NOTE: If reviewer comments were submitted as an attachment file, they will be attached to this email and accessible via the submission site. Please log into your account, locate the manuscript record, and check for the action link "View Attachments". If this link does not appear, there are no attachment files.]

To ensure your figures meet our technical requirements, please review our figure guidelines: https://journals.plos.org/plosone/s/figures

You may also use PLOS’s free figure tool, NAAS, to help you prepare publication quality figures: https://journals.plos.org/plosone/s/figures#loc-tools-for-figure-preparation.

NAAS will assess whether your figures meet our technical requirements by comparing each figure against our figure specifications.

# Author comment: Author response to Decision Letter 2

**DOI:** 10.1371/journal.pone.0355968.r004

26 Jul 2026

Please see the attached "Response to Reviewers" document. We have carefully addressed all comments from the Academic Editor and reviewers, and revised the manuscript accordingly.

Attachment Submitted filename: renamed_c2a4f.docx

# Peer review: Decision Letter 2

**DOI:** 10.1371/journal.pone.0355968.r005

**Authors:** Tomohiro Yasuda

29 Jul 2026

Association between serum uric acid and muscle function in underweight young women: An exploratory cross-sectional study

PONE-D-26-29565R2

Dear Dr. Shinobu Sugihara,

We’re pleased to inform you that your manuscript has been judged scientifically suitable for publication and will be formally accepted for publication once it meets all outstanding technical requirements.

Within one week, you’ll receive an e-mail detailing the required amendments. When these have been addressed, you’ll receive a formal acceptance letter and your manuscript will be scheduled for publication.

An invoice will be generated when your article is formally accepted. Please note, if your institution has a publishing partnership with PLOS and your article meets the relevant criteria, all or part of your publication costs will be covered. Please make sure your user information is up-to-date by logging into Editorial Manager at Editorial Manager® and clicking the ‘Update My Information' link at the top of the page. For questions related to billing, please contact billing support.

If your institution or institutions have a press office, please notify them about your upcoming paper to help maximize its impact. If they’ll be preparing press materials, please inform our press team as soon as possible -- no later than 48 hours after receiving the formal acceptance. Your manuscript will remain under strict press embargo until 2 pm Eastern Time on the date of publication. For more information, please contact onepress@plos.org.

Kind regards,

Tomohiro Yasuda, Ph.D.

Academic Editor

PLOS One

Additional Editor Comments (optional):

Reviewers' comments:

Reviewer's Responses to Questions

**Comments to the Author**

Reviewer #1: All comments have been addressed

**********

2. Is the manuscript technically sound, and do the data support the conclusions?

Reviewer #1: Yes

**********

3. Has the statistical analysis been performed appropriately and rigorously?

Reviewer #1: Yes

**********

4. Have the authors made all data underlying the findings in their manuscript fully available?

The PLOS Data policy

Reviewer #1: Yes

**********

5. Is the manuscript presented in an intelligible fashion and written in standard English?

Reviewer #1: Yes

**********

Reviewer #1: Comments to the Author:

We thank you for your careful and extensive revision.

The authors have revised the manuscript carefully according to the reviewer's comments. The manuscript will be acceptable.

**********

what does this mean?). If published, this will include your full peer review and any attached files.

If you choose “no”, your identity will remain anonymous but your review may still be made public.

**Do you want your identity to be public for this peer review?**  For information about this choice, including consent withdrawal, please see our Privacy Policy

Reviewer #1: No

**********

# Editor report: Acceptance letter

**DOI:** 10.1371/journal.pone.0355968.r006

**Authors:** Tomohiro Yasuda

PONE-D-26-29565R2

PLOS One

Dear Dr. Sugihara,

I'm pleased to inform you that your manuscript has been deemed suitable for publication in PLOS One. Congratulations! Your manuscript is now being handed over to our production team.

At this stage, our production department will prepare your paper for publication. This includes ensuring the following:

* All references, tables, and figures are properly cited

* All relevant supporting information is included in the manuscript submission,

* There are no issues that prevent the paper from being properly typeset

You will receive further instructions from the production team, including instructions on how to review your proof when it is ready. Please keep in mind that we are working through a large volume of accepted articles, so please give us a few days to review your paper and let you know the next and final steps.

Lastly, if your institution or institutions have a press office, please let them know about your upcoming paper now to help maximize its impact. If they'll be preparing press materials, please inform our press team within the next 48 hours. Your manuscript will remain under strict press embargo until 2 pm Eastern Time on the date of publication. For more information, please contact onepress@plos.org.

You will receive an invoice from PLOS for your publication fee after your manuscript has reached the completed accept phase. If you receive an email requesting payment before acceptance or for any other service, this may be a phishing scheme. Learn how to identify phishing emails and protect your accounts at https://explore.plos.org/phishing.

If we can help with anything else, please email us at customercare@plos.org.

Thank you for submitting your work to PLOS ONE and supporting open access.

Kind regards,

PLOS ONE Editorial Office Staff

on behalf of

Prof. Tomohiro Yasuda

Academic Editor

PLOS One
